# Disseminated cryptococcosis involving the adrenal gland and brain in an immunocompetent patient: A case report

**DOI:** 10.1097/MD.0000000000045923

**Published:** 2025-11-14

**Authors:** Qinpan Rao, Fang Wu, Dongying Su, Xia Song, Jinzhan Su, Linying Ma, Jianxia Xu, Shufeng Fan, Jinwang Zhu, Jianmin Wen

**Affiliations:** a Department of Radiology, The Second Affiliated Hospital of Zhejiang University of Traditional Chinese Medicine, Hangzhou, Zhejiang, China; b Department of Radiology, The Second Area of Air Force Healthcare Center for Special Services Hangzhou, Hangzhou, Zhejiang, China.

**Keywords:** adrenal cryptococcosis, adrenalectomy, case report, disseminated cryptococcosis, imaging manifestations

## Abstract

**Rationale:**

Cryptococcosis is a fungal infection that primarily occurs in immunocompromised individuals. This infection typically affects the lungs and central nervous system, while adrenal involvement is relatively uncommon. Our research reports a rare case of adrenal cryptococcosis in a patient with normal immune function, with suspected involvement of the nervous system.

**Patient concerns:**

A 51-year-old male patient with normal immune function presented with progressive symptoms of dizziness, palpitations, and fatigue, which had persisted for 2 years. Imaging studies revealed a hypoechoic mass in the left adrenal gland, multiple intracranial lesions, and progressive hydrocephalus. The adrenal mass was initially suspected to be a tumor, but pathological examination following surgical resection confirmed a Cryptococcus infection. Despite normal endocrine and immune markers, central nervous system dissemination was strongly suspected based on the progressive changes observed on magnetic resonance imaging. The patient was treated with a combination of amphotericin B and flucytosine, leading to significant improvement in the neurological symptoms.

**Diagnoses:**

The diagnosis of adrenal cryptococcosis was confirmed through surgical pathology, and based on the results of cranial magnetic resonance imaging, it is considered that the adrenal cryptococcosis may be accompanied by potential involvement of the nervous system.

**Interventions and outcomes:**

After the diagnosis, a treatment plan was developed, and following treatment, the patient’s dizziness symptoms improved, leading to their subsequent discharge.

**Lessons:**

These results suggest that clinicians should broaden their diagnostic thinking by combining advanced imaging techniques with thorough clinical evaluation to enhance their diagnostic ability for adrenal cryptococcosis, thereby preventing missed or misdiagnoses and ensuring timely, accurate, and effective treatment to improve patient outcomes.

## 1. Introduction

Cryptococcosis is an infection caused by spherical, encapsulated, yeast-like fungi, which primarily affects individuals with compromised immune function, such as patients with *HIV*/AIDS, those with hematological malignancies, severe liver diseases, or individuals undergoing chemotherapy or immunosuppressive therapy.^[1]^ Generally, *Cryptococcus* spreads from the primary infection site to organs such as the lungs, central nervous system (CNS), skin, liver, spleen, lymph nodes, and pancreas.^[2]^ Given that its symptoms lack specificity and the imaging features are often not prominent, diagnosis is challenging. Cryptococcosis is generally diagnosed through imaging examinations, histopathology, antigen detection, procalcitonin, or culture of specimens from the infection sites of immunocompromised hosts, The use of CGB (L-canavalia protein, glycine, and bromocresol blue) agar medium or matrix-assisted laser desorption/ionization time-of-flight mass spectrometry for *Cryptococcus* species identification will contribute to precise clinical diagnosis and treatment.^[3]^ The primary treatment comprises antifungal therapy using amphotericin and flucytosine.^[4]^ However, if is no significant change in lesions after a period of antifungal drug treatment, surgery may be considered as an alternative treatment strategy.

In developed countries, the most common cause of adrenal infection is autoimmune adrenalitis. In contrast, in developing countries, *Mycobacterium tuberculosis* is the leading trigger of adrenal infections. In elderly patients and those with compromised immunity, opportunistic fungal infections such as histoplasmosis, coccidioidomycosis, and blastomycosis are particularly common causes of adrenal infection.^[5]^ Cryptococcus is a rare cause of adrenal infection, especially in immunocompetent patients, with only a few cases reported in the literature.^[2,3]^ This report describes a novel case of adrenal cryptococcosis in an immunocompetent patient, characterized by magnetic resonance imaging (MRI) findings of progressive hydrocephalus and multiple abnormal signal foci in the brain, suggestive of neurological involvement. Herein, we present a brief review of the cases of adrenal cryptococcal infection reported in the literature, summarize the current imaging manifestations and pathophysiological knowledge of this disease, and discuss the diagnostic strategies for these cases.

## 2. Case presentation

**Figure 1.**
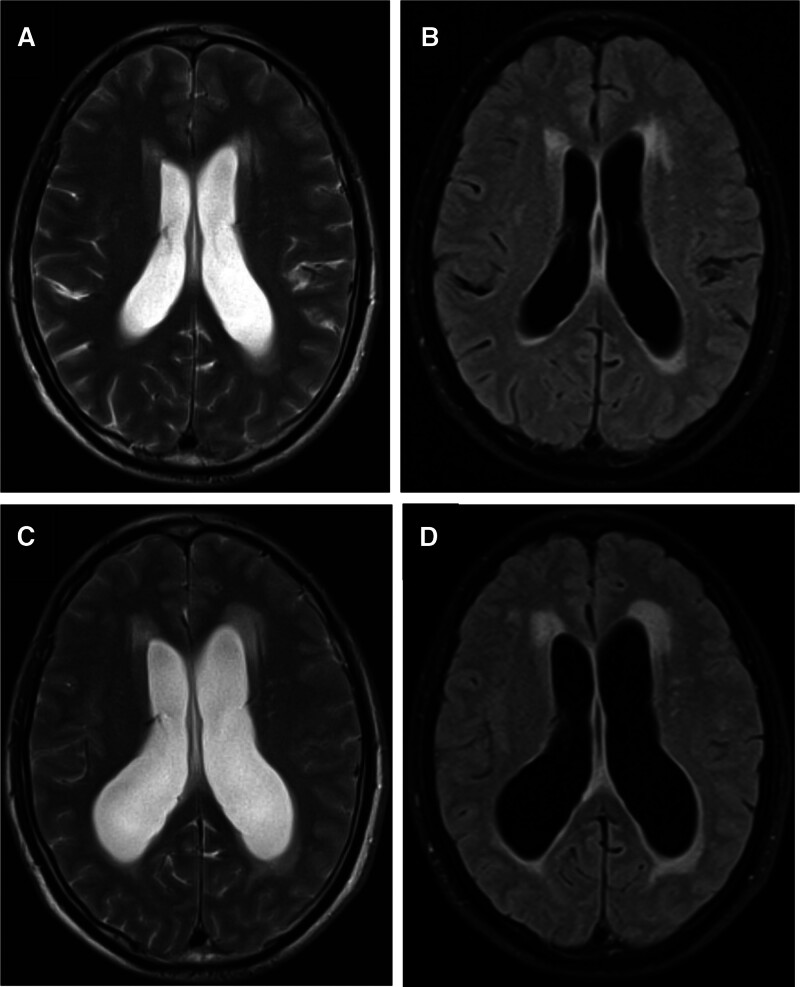
Cranial magnetic resonance imaging (MRI) of the patient. (A and B) Images of January 2018; (C and D) images of December 2019. (A) and (C) T2WI; (B) and (D) T2WI-Flair. (A and B) Cranial MRI images of the patient in 2018 showed mild hydrocephalus and a few high-signal intensities in the periventricular white matter. (C and D) Cranial MRI images of the patient in 2019 demonstrated progression of hydrocephalus. Compared to 2018 and an increase in the number of high-intensity signals within the cranium.

A 51-year-old male factory worker initially presented to our hospital in December 2018 with a primary complaint of persistent dizziness. At the first visit, his blood pressure was recorded as 170/96 mm Hg. Cranial MRI revealed mild hydrocephalus accompanied by scattered hyperintense white matter lesions in the bilateral periventricular regions, basal ganglia, and centrum semiovale (Fig. 1A and B). The mild hydrocephalus is considered to be caused by age-related changes in an elderly female, with widening of the sulci and fissures and shrinking of the gyral volume. Active follow-up was arranged for management. Additionally, the patient has stage 2 hypertension, and cranial MRI showed hyperintensity in the white matter, which was thought to be due to hypertensive small vessel disease, with demyelination, axonal injury, and glial proliferation observed in the white matter. Consequently, aggressive blood pressure management was implemented at that time. The patient was initiated on oral levamlodipine besylate 2.5 mg once daily for blood pressure control and discharged with instructions for regular medication adherence.

In September 2019, the patient returned with aggravated dizziness despite self-reported compliance with antihypertensive therapy over the preceding year. Notably, he developed concomitant symptoms including palpitations and generalized fatigue. On readmission, his blood pressure measured 155/99 mm Hg. Follow-up cranial MRI indicated aggravation of both the hydrocephalus and white matter hyperintensity. To rule out secondary hypertension caused by adrenal lesions, computed tomography (CT) and enhanced MRI of the adrenal glands were performed, revealing a left adrenal mass. Physical examination demonstrated stable vital signs without fever, upper respiratory tract infection manifestations, or neurological deficits. The medical history was unremarkable for familial disorders, psychiatric conditions, or prior surgical interventions.

### 2.1. Imaging examination

**Figure 2.**
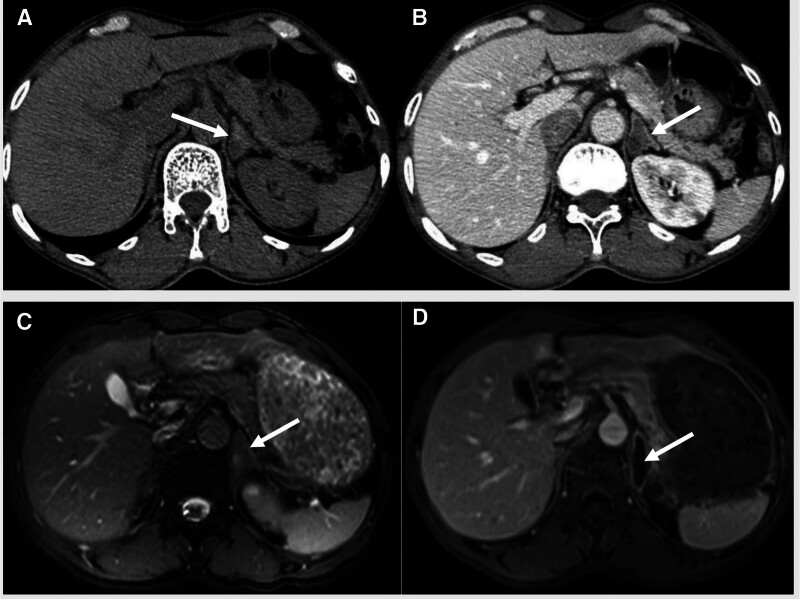
Enhanced computed tomography (CT) and magnetic resonance imaging (MRI) of the patient’s adrenal glands. (A) CT non-enhanced scan; (B) CT enhanced scan; (C) T2WI of MRI; (D) MR enhanced scan. MRI = magnetic resonance imaging.

In December 2019, a follow-up head MRI demonstrated progressive hydrocephalus and multiple patchy abnormal signal foci in the bilateral basal ganglia region, adjacent to the lateral ventricles, and in the white matter of the frontal and parietal lobes. Compared with the cranial MRI performed in January 2018 (which had shown mild hydrocephalus and a few scattered hyperintense white matter lesions), the hydrocephalus had markedly progressed, and the number of abnormal signal foci in the brain had significantly increased (Fig. 1C and D). In November 2019, enhanced adrenal CT and MRI showed an enlarged left adrenal gland. A 2.5 cm × 1.7 cm mass was identified in the body of the left adrenal gland. The medial branch of the right adrenal gland was thickened. The enhanced scan showed no significant enhancement, and presented low enhancement compared with the normal adrenal gland (Fig. 2A–D).

### 2.2. Laboratory examination

Hematological analysis revealed elevated white blood cell count, neutrophil count, and C-reactive protein level, erythrocyte sedimentation rate and procalcitonin level were within the normal range. Endocrinological assessment indicated that serum cortisol, 24-hour urinary cortisol, adrenocorticotropic hormone, angiotensin II, plasma renin activity, and aldosterone levels were all within the normal range, demonstrating intact adrenal endocrine function. Immunological examinations showed normal HIV serology results, immunoglobulin levels, and counts of CD3+ T cells and CD4+ T cells. Additionally, serum levels of potassium, sodium, creatinine, and glucose, as well as liver function parameters, tumor markers, and T-cell subset analysis results, were also within the normal range (Table 1). Through detailed medical history taking, it was confirmed that the patient had no history of long-term (exceeding 2 weeks) use of glucocorticoids via oral, intravenous, or topical routes within the past 5 years, nor had the patient used biological agents such as tumor necrosis factor antagonists or immune checkpoint inhibitors.

**Table 1:** Analysis results of patient hematology, endocrinology, and immune function.

Item	Parameter	Value	Normal range
Blood test	White blood cell	14.5 × 10^9^/L	3.5–9.5
	Neutrophil	88.9%	40.0–75.0
	Lymphocyte	15.0%	20.0–50.0
	CPR	67.18 mg/L	0–10
	ESR	16.2 mm/h	0–20
	PCR	0.02 ng/mL	≤0.05
Hormone	24-hour urine cortisol	200.18 µg/24 h	19.30–317.50
	Serum cortisol	68.89 µg/L	34.40–167.60
	Adrenocorticotropic hormone	11.38 pmol/L	1.6–13.9
	Aldosterone (upright position)	261.42 ng/L	Normal diet, upright position: 50.00–313.00 Low sodium, upright position: 60.00–650.00
	Angiotensin II (supine position)	54.38 ng/L	Normal diet: 23.00–75.00 Low sodium: 30.00–60.00
	Plasma renin activity (supine position)	0.52 µg/L/h	Normal diet, supine position: 0.13–1.74 Low sodium, supine position: 0.60–1.50
Immune function	IGG immunoglobulin	10.70 g/L	7.51–15.60
	IGM immunoglobulin	0.88 g/L	0.82–4.53
	IGM immunoglobulin	0.66 g/L	0.46–3.04
	C3 complement	0.84 g/L	0.79–1.52
	C4 complement	0.22 g/L	0.16–0.38
T-cell subset analysis	CD4+ T	856/µL	410–1590
	CD8 + T	523/µL	190–1140
	CD4+/CD8+ ratio	1.64	0.71–2.78
BG	FBG	5.3 mmol/L	3.9–6.1mmol/L
	HbA1c	5.6%	4.0–6.0%
Liver function	ALT	23 µ/L	9–50
	AST	21 µ/L	15–40
Tumor markers	CEA	2.2 ng/mL	0–5.0
	AFP	3.5 ng/mL	0–8.0
	CA199	5.7 µ/mL	0–37.0
	CA125	10.2 µ/mL	0–35.0

AFP = alpha-fetoprotein, ALT = alanine aminotransferase, AST = aspartate aminotransferase, BG = blood glucose, CEA = carcinoembryonic antigen, CPR = C-reactive protein, ESR = erythrocyte sedimentation rate, FBG = fasting blood glucose, IGG = immunoglobulin G, IGM = immunoglobulin M, PCR = procalcitonin.

### 2.3. Surgical outcomes and treatment

The patient underwent unilateral adrenalectomy, during which a tumor measuring 3.0 × 2.0 cm was detected between the medial and lateral branches of the left adrenal gland. The decision to proceed with unilateral adrenalectomy was made after multidisciplinary consultation. Although percutaneous biopsy was considered as a less invasive diagnostic option, it was ultimately deemed that surgical resection would be the most definitive management strategy. This decision was based on several factors: the significant size of the left adrenal mas with imaging features that were suspicious for a neoplastic lesion; the potential risk of needle tract seeding or hemorrhage if the lesion was indeed a neoplastic lesion; and the therapeutic benefit of complete resection, which would serve both diagnostic and curative purposes simultaneously. Given the patient’s preserved adrenal function and the low risk of adrenal insufficiency following unilateral resection, surgery was considered a safe and efficient approach to achieve a definitive diagnosis and treatment.

**Figure 3.**
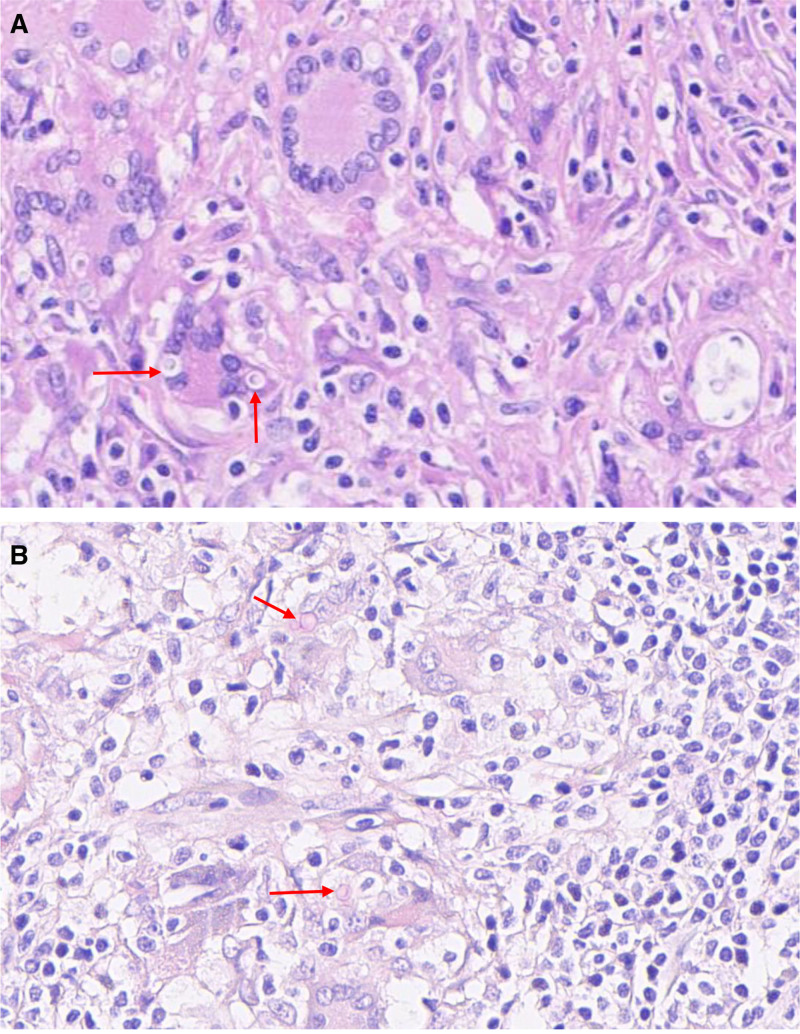
Pathological images of the patient. (A) HE stain. Under high magnification, multinucleated giant cells are visible (indicated by the red arrows). Cryptococcus bodies are present in the cytoplasm, which are round in shape and surrounded by transparent spaces. (B) Mucicarmine stain. Cryptococcal bodies are seen in the cytoplasm, and the transparent spaces around the fungal bodies are stained red. HE = hematoxylin–eosin.

Histopathological analysis revealed granulomatous inflammation with extensive coagulative necrosis. Hematoxylin–eosin staining demonstrated numerous round-to-oval yeast-like organisms (4–10 μm in diameter) within multinucleated giant cells and histiocytes, each surrounded by a distinct clear halo (capsular space) indicative of the polysaccharide capsule characteristic of Cryptococcus (Fig. 3A, red arrows). Critically, mucicarmine staining confirmed the diagnosis by exhibiting intense carmine-red coloration of the fungal capsules, pathognomonic for acid mucopolysaccharides unique to Cryptococcus species (Fig. 3B). The diagnosis of adrenal cryptococcosis was subsequently established. As treatment, amphotericin B deoxycholate (0.7 mg/kg/d) was combined with flucytosine (100 mg/kg/d, given in 4 divided administrations). The planned induction therapy spanned a total of 6 weeks. For the final 4 weeks of this induction phase, liposomal amphotericin B was administered, consistent with guideline recommendations.^[1]^ Renal function (serum creatinine) and electrolytes (potassium and magnesium) were monitored daily during the first week and 3 times per week thereafter, remaining stable throughout the course. At the outset of treatment, the patient experienced mild dizziness but had no blurred vision, headache, convulsions, or other discomforts. No additional drug intervention was required. After 1 week, the patient’s dizziness gradually improved. The patient exhibited good drug tolerance with no serious adverse reactions requiring treatment discontinuation. After the initial 6-week induction phase, the patient was discharged in stable condition and prescribed fluconazole capsules (400 mg once daily) for an additional 8 weeks as consolidation therapy. Two months after discharge, a phone inquiry about the patient’s fluconazole capsule use revealed that the patient had been taking the medication regularly as per the prescription. The patient was advised to monitor her blood pressure and follow up if any discomfort occurred. Six months later, during a follow-up call, the patient reported improvement in her symptoms, with occasional mild dizziness, but no chest tightness or fatigue. She was advised to continue monitoring her blood pressure. This content has been incorporated into the manuscript and highlighted in red.

## 3. Discussion

Adrenal cryptococcosis is relatively rare. Symptoms of primary adrenal insufficiency, such as fatigue, anorexia, hyponatremia, hyperkalemia, and skin pigmentation, more commonly seen in immunocompromised patients.^[6–8]^ In patients with normal immune function and adrenal cryptococcosis, adrenal cortical function is typically preserved. Although the patient in this case presented with fatigue, both their immune function and adrenal function markers indicated that the symptom was nonspecific in nature.

Although most cases of disseminated cryptococcosis occur in individuals with compromised immune systems, rare cases may also arise in immunocompetent individuals. Among various related factors, the acquisition of autoantibodies against granulocyte-macrophage colony-stimulating factor (GM-CSF) has been identified as a significant trigger for cryptococcal infection.^[9]^ GM-CSF plays a crucial role in maintaining normal immune function, particularly in the function of alveolar macrophages. When autoantibodies against GM-CSF are produced, they neutralize endogenous GM-CSF, leading to impaired function of alveolar macrophages, which decreases their ability to phagocytize and eliminate pathogens, as well as to initiate immune responses. In this scenario, cryptococci can bypass the host defenses and lead to infections that may progress into disseminated disease.^[10,11]^

Early diagnosis and effective monitoring of disease activity are crucial for the optimal management of fungal infections. Currently, reports on the radiological features of adrenal cryptococcosis in the literature are limited. In this case, a CT scan of the adrenal glands showed thickening of the right adrenal gland and a mass in the left adrenal gland. The left adrenal mass occupied a large area of the gland and presented with heterogeneous density, showing areas of both high and low signal intensity. MRI further revealed an uneven signal on T2-weighted images, with no significant enhancement post-contrast. Compared to healthy adrenal tissue, the mass showed significant hypo-enhancement characteristics. The imaging features of the medial branch of the right adrenal gland were consistent with the enlargement of the left adrenal gland, in accordance with previous reports.^[12,13]^ Adrenal adenomas typically present as unilateral lesions and exhibit marked arterial-phase enhancement with washout during the venous phase; however, the imaging features in this case did not conform to this pattern.

The differential diagnosis for an adrenal mass with concomitant CNS lesions in an immunocompetent patient importantly includes tuberculosis and histoplasmosis, both of which can present with granulomatous inflammation on histopathology. Tuberculosis was considered less likely due to the absence of classic histological features such as caseating granulomas with Langhans giant cells and negative acid-fast bacilli staining of the adrenal tissue specimen. Histoplasmosis, although a possibility, was effectively ruled out by histopathological findings. Mucicarmine staining revealed bright carmine-red coloration of the fungal capsules, pathognomonic for the acid mucopolysaccharide capsule of Cryptococcus species. In contrast, Histoplasma capsulatum is typically smaller (2–4 μm), exhibits narrow-based budding, and lacks a mucicarmine-staining capsule. The combination of histological appearance and specific staining patterns was conclusive for cryptococcosis and against these other fungal and mycobacterial pathogens.

This misdiagnosis case highlights the importance of considering fungal infections when clinical presentations and imaging features do not align with common diseases. The radiological characteristics of adrenal cryptococcosis have high specificity. For example, caseous necrosis does not enhance after contrast administration, which helps distinguish it from other neoplastic lesions. Although a definitive diagnosis still requires histopathological examination, imaging plays a crucial role in differential diagnosis, assessing the extent of the disease, monitoring treatment efficacy, and detecting recurrence.

Under normal circumstances, Cryptococcus infects the respiratory tract, causing pneumonia in immunocompromised patients. It can subsequently cross the blood-brain barrier, leading to meningitis, meningoencephalitis, or space-occupying brain lesions, often accompanied by increased intracranial pressure and hydrocephalus.^[14–17]^ At this stage, patients may develop neurological sequelae such as fever, altered mental status, meningeal irritation, headache, lethargy, coma, and ultimately death.^[18–20]^ In immunocompetent individuals, latent fungal infections may develop asymptomatically and disseminate to other tissues upon subsequent immunosuppression. In our case, the patient did not exhibit typical symptoms such as fever or headache, aside from dizziness. MRI revealed multiple abnormal signal foci in the brain and hydrocephalus. Compared to MRI performed 1 year prior, the number of abnormal signal foci had significantly increased, and hydrocephalus had markedly progressed. These intracranial imaging findings were highly inconsistent with the patient’s age and medical history. Although lumbar puncture was not performed due to the patient’s reluctance and clinical stability, the histopathological confirmation of adrenal cryptococcosis, combined with the characteristic brain imaging abnormalities and significant symptomatic improvement following antifungal therapy, strongly suggests intracranial cryptococcal dissemination. The failure to consider fungal infection during the initial diagnostic process underscores the necessity of maintaining a broad differential diagnosis in complex clinical and imaging scenarios. Following antifungal treatment, the patient’s dizziness improved, and he achieved a favorable outcome during follow-up.

This study has several limitations. Firstly, lumbar puncture was not performed to obtain cerebrospinal fluid for analysis due to the patient’s reluctance, thus precluding definitive microbiological confirmation of CNS involvement, such as cerebrospinal fluid cryptococcal antigen testing or culture. Secondly, fungal culture and species identification (e.g., to differentiate between *Cryptococcus neoformans* and *Cryptococcus gattii*) were not conducted on the resected adrenal specimen postoperatively. Thirdly, according the European Organisation for Research and Treatment of Cancer, further tests such as CGB (Lentil Lectin, Glycine, Bromocresol Blue), agar culture medium, matrix-assisted laser desorption/ionization time-of-flight mass spectrometry, or cerebrospinal fluid puncture biopsy should be required. Unfortunately, in this case, these tests were not conducted, and this may lead to some uncertainty in the results, such as whether the cerebral changes are caused by cryptococcal dissemination from the adrenal gland. Consequently, the potential influence of the specific cryptococcal species on the disease presentation in this immunocompetent host remains unexplored. Future studies should prioritize these diagnostic steps to enhance the understanding of similar cases.

## 4. Conclusion

This case underscores that cryptococcosis, albeit rare, can manifest as adrenal involvement with potential neurological dissemination even in immunocompetent hosts. Diagnostic challenges arise from nonspecific clinical presentations and imaging features overlapping with tumors or other infections, necessitating histopathological confirmation through characteristic findings. Clinicians should maintain a high index of suspicion in atypical cases, prioritize multidisciplinary integration of imaging and pathology, and enhance diagnostic vigilance for rare entities to mitigate delays in targeted antifungal therapy.

## Acknowledgments

The authors thank Editage for translating the text into English.

## Author contributions

**Data curation:** Qinpan Rao, Dongying Su, Xia Song, Jinzhan Su, Jianxia Xu, Shufeng Fan, Jinwang Zhu, Jianmin Wen.

**Formal analysis:** Qinpan Rao, Fang Wu, Dongying Su, Xia Song, Jinwang Zhu.

**Funding acquisition:** Qinpan Rao.

**Investigation:** Fang Wu, Dongying Su, Xia Song, Linying Ma.

**Methodology:** Jinzhan Su, Linying Ma, Jianxia Xu, Jianmin Wen.

**Supervision:** Jinzhan Su.

**Validation:** Jinwang Zhu.

**Writing – original draft:** Qinpan Rao.

**Writing – review & editing:** Shufeng Fan, Jianmin Wen.
